# Reduced Nasal Nitric Oxide Production in Cystic Fibrosis Patients with Elevated Systemic Inflammation Markers

**DOI:** 10.1371/journal.pone.0079141

**Published:** 2013-11-13

**Authors:** Ruth K. Michl, Julia Hentschel, Christiane Fischer, James F. Beck, Jochen G. Mainz

**Affiliations:** Department of Paediatrics, Jena University Hospital, Jena, Germany; University of Colorado, Denver, United States of America

## Abstract

**Background:**

Nitric oxide (NO) is produced within the respiratory tract and can be detected in exhaled bronchial and nasal air. The concentration varies in specific diseases, being elevated in patients with asthma and bronchiectasis, but decreased in primary ciliary dyskinesia. In cystic fibrosis (CF), conflicting data exist on NO levels, which are reported unexplained as either decreased or normal. Functionally, NO production in the paranasal sinuses is considered as a location-specific first-line defence mechanism. The aim of this study was to investigate the correlation between upper and lower airway NO levels and blood inflammatory parameters, CF-pathogen colonisation, and clinical data.

**Methods and Findings:**

Nasal and bronchial NO concentrations from 57 CF patients were determined using an electrochemical analyser and correlated to pathogen colonisation of the upper and lower airways which were microbiologically assessed from nasal lavage and sputum samples. Statistical analyses were performed with respect to clinical parameters (lung function, BMI), laboratory findings (CRP, leucocytes, total-IgG, fibrinogen), and anti-inflammatory and antibiotic therapy. There were significant correlations between nasal and bronchial NO levels (rho = 0.48, p<0.001), but no correlation between NO levels and specific pathogen colonisation. In patients receiving azithromycin, significantly reduced bronchial NO and a tendency to reduced nasal NO could be found. Interestingly, a significant inverse correlation of nasal NO to CRP (rho = −0.28, p = 0.04) and to leucocytes (rho = −0.41, p = 0.003) was observed. In contrast, bronchial NO levels showed no correlation to clinical or inflammatory parameters.

**Conclusion:**

Given that NO in the paranasal sinuses is part of the first-line defence mechanism against pathogens, our finding of reduced nasal NO in CF patients with elevated systemic inflammatory markers indicates impaired upper airway defence. This may facilitate further pathogen acquisition in the sinonasal area, with consequences for lung colonisation and the overall outcome in CF.

## Introduction

Cystic fibrosis (CF) is one of the most frequent autosomal recessive disorders in Caucasians. Pathogens like *Pseudomonas (P.) aeruginosa* colonising the airways cause chronic pulmonary infections with a permanent inflammation process and, ultimately, a life-limiting destruction of the lungs. Recently, the upper airways (UAW), especially the nasal cavity and paranasal sinuses have come into scientific focus as site of first and persistent airway colonisation in CF. In this regard, anatomical and immunological conditions facilitating sinonasal colonisation with pathogens are of special interest [1], [2].

In routine clinical care, efficient monitoring of pathogen colonisation, lung function, and laboratory parameters is necessary to optimize CF therapy. Currently, the standard of airway sampling in CF comprises the lower airways with sputum, throat swab, and – for special scientific and clinical questions – bronchoalveolar lavage [3]. Upper airway sampling does not belong to the current standards, but nasal lavage enables a non-invasive and repeated sampling that can be used as a supplementary diagnostic tool [2], [4], [5].

Nitric oxide (NO) is a free radical gas produced in the lower and – in markedly higher concentrations – in the upper respiratory tract, which functions as a messenger molecule. As a mediator of inflammation processes, NO affects vasodilatation and bronchodilatation, is a member of the primary upper airway defence with its antimicrobial activity, and can be detected in exhaled air [6], [7]. There are three isoforms of the NO forming enzyme, called NO synthase (NOS): neuronal NOS (NOS1), inducible NOS (NOS2), and endothelial NOS (NOS3). Whereas NOS1 and NOS3 are expressed constitutively, NOS2 is known to be upregulated by pro-inflammatory cytokines in inflammation processes [8]. Thus, despite NO concentrations being variable in exhaled air, it is generally increased within inflammatory lung diseases like asthma and bronchiectasis [9], [10]. The upper airways and, specifically, the paranasal sinuses are the major source of NO [11]. In primary ciliary dyskinesia, nasal NO is reduced consistently [7]. Therefore, assessment of exhaled nasal NO is used as a non-invasive diagnostic test for this inherited disease [6], [12]. The bronchial NO levels in CF patients, typically having a chronic inflammatory lung disease, are, surprisingly, equal [10], [13], [14] or even decreased [13], [15], [16] in comparison to healthy control groups. Nasal NO levels are known to be lower in CF patients than in control subjects [10], [13], [14]. The aim of this study was to quantify NO in exhaled bronchial and nasal air and to correlate the NO concentrations to CF-pathogen colonisation as well as to clinical and laboratory parameters in order to collect more information on differences in upper and lower airway inflammatory reactions and NO production.

## Patients, Materials, and Methods

57 patients from the Jena University Hospital CF outpatient clinic were enrolled in the study between August 2010 and January 2012. Inclusion criterion for patients was diagnosis of CF: confirmed by at least two positive sweat tests and/or two CFTR disease-causing mutations. Patients aged less than five years were excluded from the study due to difficulties or experimental uncertainties with NO measurements usually occurring with smaller children. There were 26 males and 31 females aged 5–73 years (mean age 20.9 years), 28 adults, and 29 children. Details of the study population are given in Tables 1 and 2.

**Table 1:** Clinical and laboratory parameters of studied CF patients.

Metric variables	n	Mean	SD	Median	Range
Age	57	20.91	12.76	21.00	5–73
Lung function FEV1 (% predicted)	56	80.14	30.88	83.00	23–128
Lung function FVC (l)	53	89.75	21.93	92.00	41–127
BMI (kg/m^2^)	57	18.95	2.83	18.50	13.2–26.0
BMI percentile	26	46.00	23.85	44.00	2–89
CRP (mg/l)	54	5.52	7.58	2.00	2–33
Leucocytes (Gpt/l)	53	8.69	3.30	8.00	4.2–22.3
IgG (g/l)	40	12.08	4.52	11.15	5.1–23.2
Fibrinogen (g/l)	51	2.98	0.52	2.90	2.2–4.4

Abbreviations: SD – standard deviation; FVC – forced vital capacity; FEV 1– forced expiratory volume in one second; BMI – body mass index; CRP – C-reactive protein; IgG – immunoglobulin G.

**Table 2:** Pathogen colonisation and therapy of studied CF patients.

Nominal variables	n	Absolute frequency	Relative frequency
Permanent colonization LAW:	57		
*- P. aeruginosa*		21	36.8%
Detection in UAW:	57		
*- P. aeruginosa*		8	14.0%
*- S. aureus*		21	36.8%
Detection in LAW:	57		
*- P aeruginosa*		19	33.3%
*- S. aureus*		21	36.8%
Current steroid therapy:	57		
- nasal		16	28.1%
- bronchial		22	38.6%
Current inhalative antibiotic therapy	57	31	54.4%
Current azithromycin therapy	57	19	33.3%

Abbreviations: LAW – lower airways; UAW – upper airways; *P. aeruginosa* – *Pseudomonas aeruginosa*; *S. aureus* – *Staphylococcus aureus*.

### Ethics

This retrospective study strictly complied with the guidelines of the Declaration of Helsinki and was approved by the ethics committee of the Jena University Hospital (registration number 2909-08/10). All patients (or parents of minors) gave their written informed consent.

### Microbiology

Sputum and nasal lavage (NL) were collected for microbiologic analyses, in order to assess material from both the nasal cavity (UAW) and the lungs (LAW). If a patient was not able to produce sputum or perform NL, respiratory and/or nasal swab samples were obtained. Sputum samples were taken according to current standards. Additional non-invasive upper airway sampling was collected by diagnostic NL.

Diagnostic NL was performed as described previously [2], [17]. In brief, 10 ml of isotonic saline was slowly instilled into each nostril, using a 10 ml syringe, while the patient was reclining the head and closing the soft palate. The solution was retained for approximately 10 seconds in the nasal cavity and afterwards expulsed into a sterile plastic beaker by forwarding and flexing the head.

*P. aeruginosa* serum antibodies for elastase, exotoxin A, and alkaline protease were assessed by Mediagnost, Reutlingen, Germany.

### Statistical Analysis

Statistical analyses were performed using SPSS version 19.0 (SPSS Inc., Chicago, Ill., USA) and Prism version 6.01 (GraphPad Software Inc., La Jolla, CA, USA). Metric variables of clinical and laboratory data were usually expressed as mean together with 95% confidence intervals, complemented by range values where appropriate. Univariate nonparametric tests were chosen to compare mean values of two independent samples (Wilcoxon-Mann-Whitney test) or two and more independent groups (Kruskal-Wallis test). Correlations between variables were assessed using Spearman’s rank correlation coefficient rho.

To investigate the influence of potential confounding factors, a multivariate analysis of bronchial and nasal NO concentration was performed. Among the characteristics measured, additional clinically important variables (i.e., CRP, and forced expiratory volume in a second, FEV1) were selected and incorporated as covariates in two univariate multiple linear regression models describing the decadic logarithm of bronchial and nasal NO concentrations, respectively. Residuals were tested for normality to assess model assumptions.

All reported p-values are two-tailed and we define statistical significance below the 0.05 level.

### Nitric Oxide Measuring

NO was measured using an electrochemical NO analyser (FILT NO VARIO Analysator, Berlin, Germany) according to international guidelines [18]. Under visual control on a computer screen, patients exhaled through a mouthpiece against a positive pressure of 10 mm Hg in order to achieve a constant flow rate of 50 ml/s.

For nasal NO measurement, one nostril was closed with an inserted olive, leaving the other nostril open. Air was sampled while exhaling with the soft palate closed. Closure of the soft palate was achieved while slowly exhaling against standardized positive pressure.

Measurements were taken twice and the mean NO concentration was used for further analyses, as recommended by the American Thoracic Society (ATS) [18]. As there are conflicting results in different studies [19], [20] to whether a circadian effect on nasal NO can be found, we followed the guidelines of the ATS, recommending measurement of nasal NO at the same time of the day.

### Spirometry

Spirometry was performed with Master Screen Body (Jaeger/Toennies, Germany), FVC (forced vital capacity) and FEV1 (forced expiratory volume in a second) were expressed as percentage of predicted values. Reference values were calculated according to Polgar et al [21] for adults and Zapletal et al [22] for children.

## Results

### Correlation between Bronchial and Nasal Nitric Oxide

**Figure 1:**
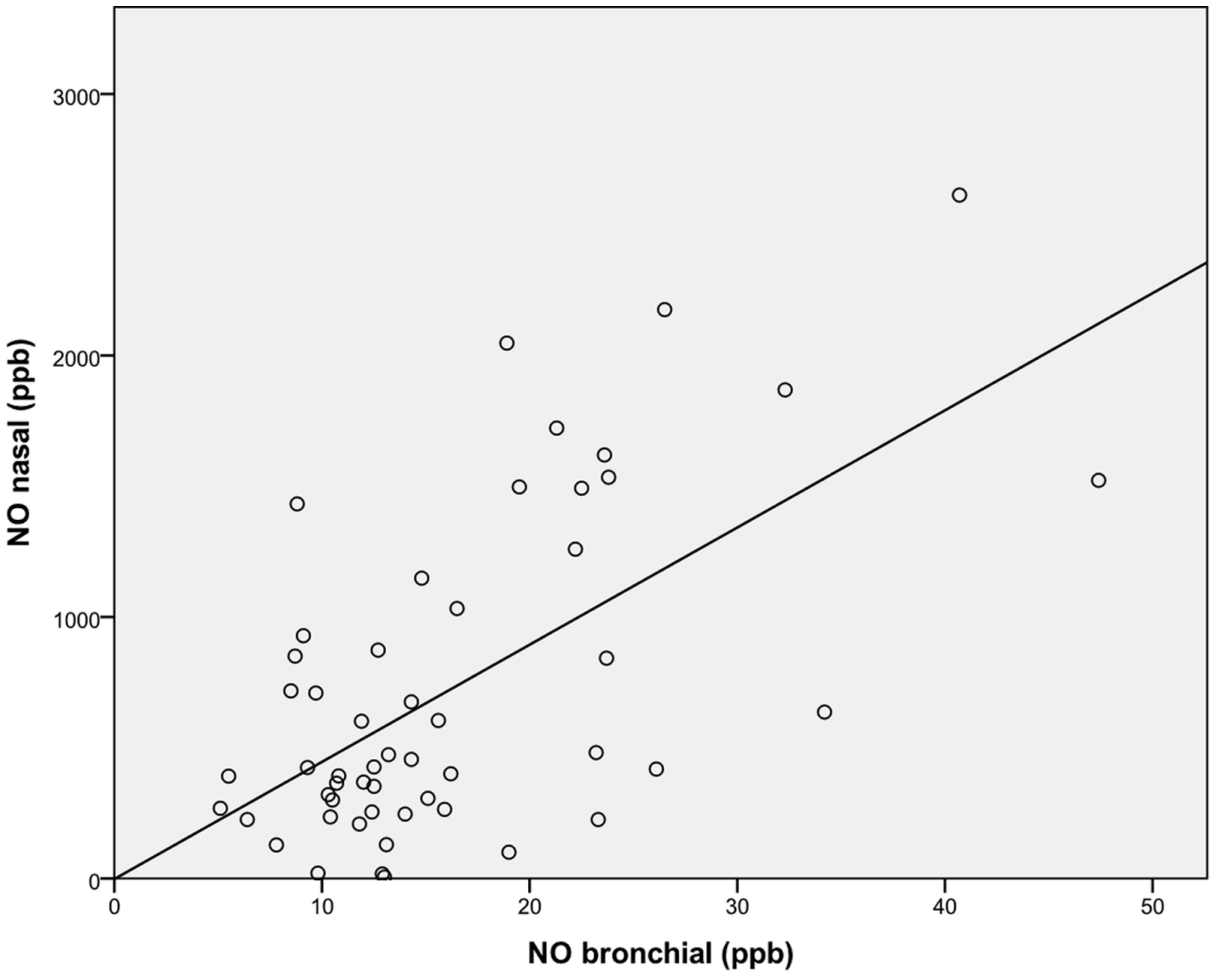
Scatter plot showing the correlation between nasal and exhaled bronchial NO levels. The solid line represents a best fit to the experimental data, assuming linear regression.

Bronchial and nasal nitric oxide measurements correlated significantly (Spearman’s correlation coefficient rho = 0.48, p<0.001, see Figure 1), despite the very different concentration levels.

### Correlation between Nitric Oxide and Clinical Parameters: Nutritional Status/Body Mass Index

The body mass index (BMI) was used as nutritional status indicator for adult patients, while BMI percentiles were used for children. Nutritional status was categorized according to the recommendation of the WHO [23]. Neither bronchial NO nor nasal NO concentrations showed significant differences between the BMI groups.

Since all enrolled patients were pancreas insufficient, a comparison between pancreas sufficient and pancreas insufficient patients was not possible.

### Correlation between Nitric Oxide and Clinical Parameters: Lung Function

The FEV1 ranged from 23% to 128% (mean value 80.1%). FVC varied between 41% and 127%, with a mean value of 89.8%.

Bronchial NO was neither correlated to predicted FEV1 (rho = 0.11, p = 0.42) nor predicted FVC (rho = 0.22, p = 0.13). Furthermore, our data doesn’t show a correlation between nasal NO and predicted FEV1 (rho = 0.17, p = 0.21) or predicted FVC (rho = 0.18, p = 0.21), and no correlation was found between age and bronchially exhaled or nasal NO, respectively.

### Correlation between Nitric Oxide and Pathogen Colonisation

We categorized patients according to pathogen colonisation in the lower airways into 3 groups with ‘no pathogen colonisation’ (meaning neither *P. aeruginosa* nor *S. aureus)*, ‘colonised with *P. aeruginosa*’, and ‘colonised with *S. aureus*’. There was neither a significant difference in bronchial nor nasal NO concentrations in those patients colonised with *P. aeruginosa* and those not colonised. The same statement held true when comparing patients colonised with *S. aureus* and those not (all respective p-values>0.35). No significant differences in lung function or age between the two pathogen colonised study groups could be seen. We assessed potential correlations between NO and pathogen colonisation once again separately for the lower and upper airways, i.e., defining patient groups according to bronchial and nasal pathogen colonisation, respectively. Still, no significant differences in bronchial/nasal NO concentrations between the pathogen colonisation groups ‘*P. aeruginosa*’, ‘*S. aureus*’, ‘both pathogens’ and ‘neither of the two’ were detected in our study.

At the time of NO sampling, 19 patients (33%) were tested *P. aeruginosa* positive in the bronchial sampling (sputum or respiratory swab), 8 (14%) were tested *P. aeruginosa* positive in the nasal sampling. 21 (37%) of the enrolled patients had a history of chronic *P. aeruginosa* colonisation according to Leeds criteria [24]. 21 patients (37%) tested bronchially positive for *S. aureus* and 21 (37%) nasally at the time of NO measurements. Two patients (3.5%) were tested nasally positive for both pathogens and 6 bronchially (10.5%). There was no colonisation with other pathogens like nontuberculous *Mycobacteria*, *A. xylosoxidans*, *S. maltophilia* and *B. cepacia* in our study group.

We also analysed our data with respect to potential correlations between measured NO values and *P. aeruginosa* serum antibodies (elastase, exotoxin A, and alkaline protease). In our data, no correlation between *P. aeruginosa* antibodies and exhaled bronchial or nasal NO could be found.

### Correlation between Nitric Oxide and Inflammatory Serum/blood Parameters

Our data reveal a highly significant negative correlation (Spearman’s correlation coefficient rho = −0.41, p = 0.003) between nasal NO and leucocytes in blood. Furthermore, there is negative correlation between nasal NO and CRP (rho = −0.28, p = 0.04).

For the other inflammatory parameters fibrinogen and total-IgG, we did not find any significant correlation with respect to nasal NO. Bronchial NO was not correlated with any of the parameters CRP, total-IgG, fibrinogen, or leucocytes.

### Correlation between Nitric Oxide and Topical Steroids

In our study cohort, use of inhaled bronchial or nasal steroids did not significantly affect bronchial or nasal NO. 12 (21%) of the patients were taking bronchially inhaled steroids, 6 (11%) were taking nasal topical steroids, 10 (18%) were taking topical steroids for both airway levels, and none used oral steroids.

### Correlation between Nitric Oxide and Antibiotic Therapy

We found bronchially exhaled nitric oxide values to be significantly raised (p-value = 0.006) in patients during on-going, oral or inhalative chronic suppressive antibiotic treatment (mean 14.7, CI 11.6–17.8) compared with patients not receiving antibiotic treatment at the time of the NO measurement (mean 23.1, CI 15.8–30.3).

**Figure 2:**
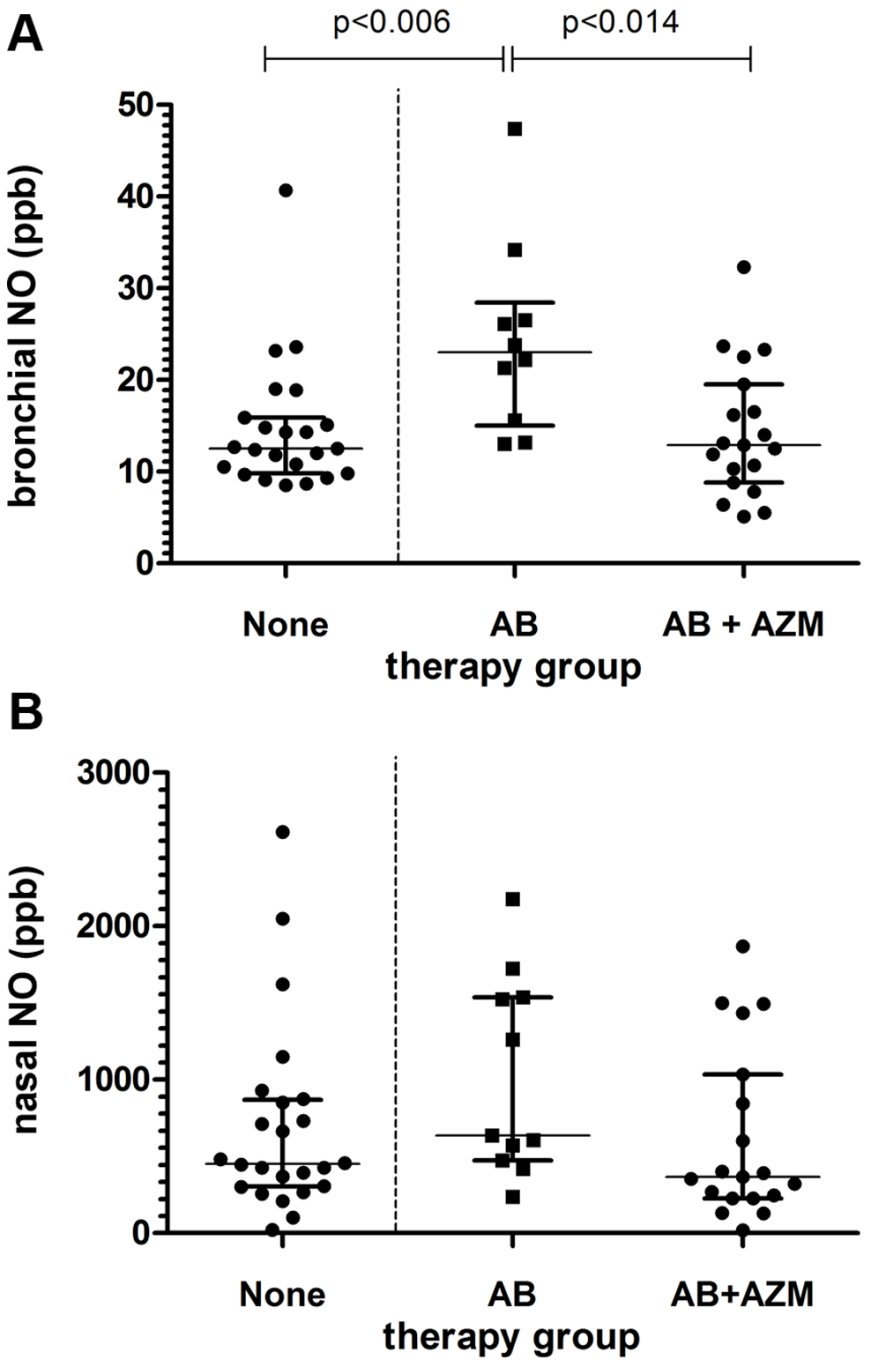
Exhaled bronchial and nasal NO concentrations. Comparison of exhaled bronchial (a) and nasal (b) NO concentrations between patients without antibiotic therapy (none), with on-going chronic suppressive antibiotic therapy (AB), and additional anti-inflammatory treatment with azithromycin (AB+AZM).

Furthermore, our data show significantly reduced bronchially exhaled NO levels for patients taking azithromycin in addition to antibiotic medication (mean 14.4, CI 10.9–17.8, p-value = 0.014), reaching NO levels very similar to those in the patient group without antibiotic therapy (Figure 2a). No patients receiving antibiotic treatment due to acute exacerbation were enrolled. Time from stopping therapeutic antibiotic treatment was at least 2 weeks.

Nasal nitric oxide measurements show the same trend, i.e., a decreased NO concentration if azithromycin is given in addition to antibiotic medication, not reaching significance, however (p-value = 0.096). For nasal NO, mean values were 694, 930, and 624 ppb, for patients receiving no antibiotic treatment, patients receiving antibiotics, and patients taking azithromycin in addition to antibiotics, respectively (Figure 2b).

A comparison between the different therapy groups showed a tendency to higher age of the patients, significantly increased frequency of colonisation with *P. aeruginosa*, and a significantly reduced FEV1 in the group with chronic suppressive antibiotic therapy and azithromycin, as expected when applying standard therapy.

Assessing the data separately for the subgroups of *P. aeruginosa* colonised (according to Leeds criteria [24]) and not colonised patients, we found the same qualitative behaviour in both subgroups as in our entire study population. Bronchial NO level differences between the therapy groups showed high significance in the *P. aeruginosa* negative patients (p-values<0.04) but not in the subgroup of *P. aeruginosa* positive patients.

### Multivariate Analysis

To verify that the individual associations found are not just incidental, multivariate analysis was performed. Taking into account the size of the presented study, we selected two categorical variables (pathogen colonisation and antibiotic use) and two metric variables (CRP and FEV1) as major influencing factors to model our observed bronchial and nasal NO concentrations. The decadic logarithm of the measured data was modelled using the linear predictor functions. To retain reasonable group sizes, pathogen colonisation was stratified into 3 groups (‘colonisation with neither *P. aeruginosa* nor *S. aureus*’, ‘colonisation with *S. aureus* but not *P. aeruginosa*’, and ‘colonisation includes *P. aeruginosa*’), and colonisation status was considered specifically in the upper and lower airways for modelling nasal and bronchial NO, respectively. Antibiotic use was categorized in the identical fashion as for univariate analysis.

For bronchial NO, multivariate analysis confirmed the earlier finding that bronchial NO is significantly reduced in the patient group receiving azithromycin in addition to standard antibiotic therapy (estimated mean value 12.6 ppb with azithromycin vs. 20.3 ppb without, p-value = 0.038). Antibiotic use was the only influencing factor reaching statistical significance, whereas pathogen colonisation, CRP, and FEV1 did not exert significant influence.

Multivariate modelling of nasal NO affirms the results of univariate analyses presented above, reproducing a negative correlation of nasal NO and CRP close to statistical significance (p = 0.053).

Table 3 summarizes the regression coefficients, p-values, and confidence intervals from multiple linear regression analysis of bronchial and nasal NO concentrations.

**Table 3:** Results of a multivariate analysis of bronchial and nasal NO concentrations, incorporating FEV1, CRP, pathogen colonisation, and antibiotic therapy into a multiple linear regression model: regression coefficients, significance levels, and confidence intervals.

Dependent variable	Explanatory variable	Regression coefficient β	p-value	95% confidence interval	
log10 NO bronchial	Constant	1.109	0.000	0.828	1.391
	bronchial colonisation with *P.a*.	0.015	0.848	−0.137	0.167
	bronchial colonisation with *S.a*.	−0.083	0.291	−0.241	0.074
	no bronchial colonisation	0			
	antibiotic therapy+Azithromycin	−0.025	0.778	−0.199	0.150
	antibiotic therapy	0.184	0.034	0.015	0.352
	no antibiotics	0			
	FEV1 (% predicted)	0.001	0.573	−0.002	0.003
	CRP (mg/l)	−0.003	0.443	−0.012	0.005
log10 NO nasal	Constant	2.576	0.000	2.106	3.046
	bronchial colonisation with *P.a*.	0.013	0.927	−0.266	0.292
	bronchial colonisation with *S.a*.	0.238	0.112	−0.058	0.534
	no bronchial colonisation	0			
	antibiotic therapy+Azithromycin	0.44	0.790	−0.285	0.373
	antibiotic therapy	0.92	0.451	−0.151	0.335
	no antibiotics	0			
	FEV1 (% predicted)	0.002	0.364	−0.002	0.006
	CRP (mg/l)	−0.015	0.053	−0.031	0.000

Abbreviations: *P.a.* – *Pseudomonas aeruginosa*; *S.a.* – *Staphylococcus aureus*; FEV 1– forced expiratory volume in one second; CRP – C-reactive protein.

## Discussion

Our study quantified and correlated upper and lower airway NO levels with CF-pathogen colonisation, blood inflammatory parameters, and clinical findings with the aim to point out similarities and differences in upper and lower airways NO production.

Despite the very different levels of NO concentration in our cohort of 57 CF patients with a wide range of age, bronchially exhaled and nasal nitric oxide measurements correlate significantly (Spearman’s correlation coefficient rho = 0.48, p<0.001). Various studies in cystic fibrosis patients have detected normal [10], [14], [25] or decreased [13], [15], [16] concentrations of bronchially exhaled NO, whereas nasal NO was shown to be lower in CF than in controls [10], [13], [14]. This finding is surprising because of the chronic inflammation in CF upper and lower airways, since NO is known to be increased during airway inflammation e.g., in asthma, bronchiectasis or respiratory tract infections [9], [10]. Nitric oxide is assumed to be upregulated by inflammatory cytokines such as TNF-α, IL-1β and interferon-γ [11], but until now, no coherence between inflammatory markers in the blood and NO could be found. Our data are in accordance with Balfour-Lynn and Keen who did not find a correlation between the NO concentration in the lower airways and clinical parameters such as lung function and weight [14], [26]. Additionally, in agreement with Keen et al [26], we could not detect any correlation between inflammatory markers in the blood (CRP, total-IgG) and bronchial NO. Several explanations have been proposed for the normal or low exhaled NO levels in CF, as well as for the lack of reaction on systemic inflammatory parameters: Firstly, the thick mucus may inhibit the diffusion of NO into exhaled air. High concentrations of nitrate and nitrite, two metabolites of NO, are found in CF airway secretions, which suggests that NO metabolites are retained in the mucus and that the exhaled fraction of NO does not reflect the total amount of NO production in the airways [27]. Secondly, a lack of the NO substrate L-arginin may contribute to low NO levels [28] in CF patients. Thirdly, the activity of the NO synthase (NOS) may be reduced in CF [29]. A fourth reason for decreased NO may be a denitrification of NO by anaerobic bacteria, e.g., through the pigment pyocyanin, which is produced by *P. aeruginosa* and inactivates NO [14], [30].

An important finding in our study is a (not previously described) negative correlation between nasal NO and CRP and a negative correlation between nasal NO and leucocytes in the blood. This is even more interesting as the inflammatory reaction should lead to an up-regulation of nitric oxide and this phenomenon could only be found in nasal NO levels.

Our findings emphasize the differences between bronchially exhaled and nasal NO and, thus, differences of the innate immune-response. One may attribute reduction of nasal NO in patients with elevated CRP to the fact that, in exacerbation, secretions in CF upper airways rise further, additionally retaining metabolites in the mucus [27] and, additionally, to the increased number of bacteria within the paranasal sinuses that may denitrify NO. For example, *P. aeruginosa* grows in an anaerobic biofilm; under such conditions, NO is used to generate energy via denitrification. Ultimately, this results in ammonium after stepwise reduction of NO to nitrite (NO2) and nitrate (NO3) [31]. Most probably, the bacterial load in the CF paranasal sinuses imply relevant consumption of NO.

The major source of NO production are the upper airways and, specifically, the paranasal sinuses are supposed to be the most important one [11]. NO has furthermore bacteriostatic activity and the paranasal sinuses are known to be first-line defence against pathogens [6]. Reduced nasal NO in CF patients with elevated CRP can give a further explanation for the role of the paranasal sinuses in acquisition and persistence of pathogens into CF airways which presently is a scientific focus [1], [32]. Obviously, in our data a substantial increase in NO levels compared to normal could not be found, so one may suggest that, due to several, up to now insufficiently studied reasons, host defence mechanisms in the upper airways are reduced. Recently, the Copenhagen CF and ENT centres stated that the defence mechanism in the nasal cavity is different from the bronchial one, but different immune response of the upper and lower airways is not yet completely understood. Aanaes et al showed a significantly higher IgA level as sign of a mucosal antibody response in the upper airways in contrast to the neutrophil dominated inflammation in the lower airways [1], [32]. Johanson et al [32] supposed the high concentrations of non-inflammatory IgA to impede the polymorphonuclear cells to be recruited, hence leading to an altered first-line defence in the upper airways in contrast to the lower airways, which are IgG dominated, promoting an inflammation via polymorphonuclear cells. In consideration of the upper airways fulfilling an important first-line defence mechanism for pathogens colonising also the lower airways [2], [33] and leading to pulmonary destruction, deeper knowledge of the immune response is of obvious importance. We have to assume that the upper airways in CF patients have an impaired defence mechanism, facilitating chronic pathogen colonisation. Further research is needed on how the metabolic activity of bacteria and inflammatory response of the patients interact, and results need to be verified in a larger number of patients.

Regarding the different pathogens colonising CF airways, our data showed no influence on bronchial or nasal NO levels (all respective p-values>0.35). We categorized patients into 3 groups with ‘no colonisation with *P. aeruginosa* or *S. aureus’*, ‘colonised with *P. aeruginosa*.’, and ‘colonised with *S. aureus’* in the lungs. In the past, conflicting results were published in this regard. Balfour-Lynn [14] found decreased bronchial NO levels in patients colonised with *S. aureus*, and decreased nasal NO levels in patients with *P. aeruginosa* colonisation, whereas our data does not support these findings. On the contrary, measured NO levels showed a tendency towards higher values for pathogen colonised patients. Other authors, e.g., Keen et al and Grasemann et al [15], [26] found lower bronchial NO levels in CF patients colonised with *P. aeruginosa*. An explanation for the different study results may be found in the fact that patients colonised with *P. aeruginosa* are more likely to have a severe course of disease and a higher complication rate. Therefore, the individual influencing factors of the patients differ considerable – consequently in the examined groups as well. We tested for a correlation between Pseudomonas antibodies in the blood, which were – to our knowledge – not examined by other studies before. Because of the antigen structure of the *P. aeruginosa* polysaccharides, infection with the pathogen leads to Pseudomonas antibodies: elastase, exotoxin A and alkaline protease. Kappler et al [34] found a sensitivity to detect colonisation of 86% and specificity of 96% in testing all three antibodies. In our study, no correlation could be found to Pseudomonas antibodies in the blood and bronchially exhaled or nasal NO.

As NO is upregulated in inflammatory response, we investigated the effect of azithromycin (AZM), which is routinely used as anti-inflammatory treatment of CF patients with chronic *P. aeruginosa* infection, on nasal and bronchial NO. Azithromycin leads to a reduction of inflammation [35], inhibits bacterial communication, and causes a reduction in neutrophil count and serum inflammatory markers proved on patients not infected with *P. aeruginosa* [35]–[37]. Additionally, azithromycin leads to a significant improvement of FEV1 and FVC in CF adults [37]. Both nasal and exhaled NO levels showed a tendency to decrease in patients (with or without *P. aeruginosa* colonisation) taking azithromycin, whereas antibiotic treatment alone had no effect, which has already been found before [25], [38]. We suppose that downregulation of inflammatory enzymes leads, via azithromycin, to downregulation of NO. Nasal nitric oxide measurements show differences, not reaching significance, however, between the treatment groups. We may assume that the paranasal sinuses in CF patients are not adequately reached by antibiotics applied by conventional inhalation or systemic application, as the effect on bronchial NO is more pronounced than on nasal NO. This is in line with a case report recently published by our group: in a patient with first and isolated sinonasal colonisation with *P. aeruginosa* [39], a two week intravenous antibiotic treatment with tobramycin and ceftazidim did not lead to eradication. As therapeutic consequence, he successfully inhaled tobramycin as vibrating aerosols for 28 days and remained free of *P. aeruginosa*. Limitations of antibiotic deposition in the paranasal sinuses can also be resolved operatively. Aanaes et al [40], [41] showed that extensive sinus surgery in combination with a strict postoperative regime consisting of i.v. antibiotics, nasal antibiotic irrigations and intensive follow up could eradicate bacteria in the upper airways of several patients for more than 1 year.

In the context of first and persistent colonisation with pathogens, the paranasal sinuses have to be seen as a focus for early airway colonisation, and the effects of an efficient therapy, especially of the upper airways, need to be evaluated to improve outcome in CF. They are of importance to prevent lung colonisation and, consequently, lung destruction in CF.

### Limitations

Interesting findings were pointed out in our study, but several limitations have to be discussed. One limiting aspect is that the compliance of the patients has not been assessed in our study regarding steroids and oral or inhalative antibiotics. Besides, with respect to microbiology, we have to assume that especially anaerobic bacteria can possibly stay undetected due to microbiology culture technique. Thus, further studies focussing on this aspect are required.

Additional important aspects such as simultaneous presence of asthma, overlap with other colonising bacteria, and variations in genotypes, need to be assessed in a larger group of patients.

### Conclusion

New interesting aspects with regard to nasal mucosal inflammation, in contrast to bronchial inflammation, as a multifactorial event are only emerging. We have to assume that the upper airways are of particular importance for pathogen defence. Hence, impaired upper airway defence adds to the vicious circle of further pathogen acquisition into the sinonasal area from where they can descent into the lower airways and cause pulmonary destruction as major reason of premature death in CF.

We believe that NO measurements may contribute to generating a deeper understanding of the delicate interplay of upper and lower airway inflammatory processes in CF.
